# CMOS-compatible metal-stabilized nanostructured Si as anodes for lithium-ion microbatteries

**DOI:** 10.1186/1556-276X-9-613

**Published:** 2014-11-14

**Authors:** Gibaek Lee, Stefan L Schweizer, Ralf B Wehrspohn

**Affiliations:** 1 Fraunhofer Institute for Mechanics of Materials IWM, Halle (Saale) 06120 Germany; 2 Department of Physics, Martin-Luther University of Halle-Wittenberg, Halle (Saale) 06099, Germany

**Keywords:** 1-D nanowires on black silicon, Lithium-ion batteries, Electrochemical characterization, Anode materials, Metal coating

## Abstract

The properties of fully complementary metal-oxide semiconductor (CMOS)-compatible metal-coated nanostructured silicon anodes for Li-ion microbatteries have been studied. The one-dimensional nanowires on black silicon (nb-Si) were prepared by inductively coupled plasma (ICP) etching and the metal (Au and Cu) coatings by successive magnetron sputtering technique. The Cu-coated nb-Si show the most promising electrochemical performance enhancements for the initial specific capacity as well as their cyclability compared to pristine nb-Si. The electrochemical and microstructural properties before and after cycling of the metal-coated nb-Si compared to their pristine counterparts are discussed in detail.

## Background

Microbatteries are required to drive small devices, such as smartcards, medical implants, and sensors. To date, the electrochemical performances of these all-solid-state batteries are limited because planar thin films are employed as electrode and electrolyte materials. The thickness of the stacking films is typically limited below 15 μm, and thus, the resulting battery reveals relatively low power and energy densities. In order to develop improved electrochemical performances, new materials and complementary metal-oxide semiconductor (CMOS)-compatible high-throughput manufacturing processes are required. Large specific area substrates by nanoarchitectured electrodes may therefore represent a promising alternative to improve the general performances of these micro power sources [1-3]. Among various anode materials in lithium-ion battery, Si has the highest theoretical specific capacity (approximately 4,200 mAh g^-1^, Li_4.4_Si), has a low Li uptake potential (approximately 0.4 V vs. Li/Li^+^), and is completely CMOS compatible [4,5]. Unfortunately, Si-based electrodes suffer from poor capacity retention caused by a large volume change (approximately 320%) of Si during Li insertion and extraction. This feature leads to cracking and pulverization of Si-based electrodes induced by the large stresses, resulting in a loss of electric contact and eventually capacity fading during cycling [6-8]. Various structural designs of Si-based electrodes have been suggested to overcome this disadvantage, such as nano- and microstructured Si, porous Si, and Si nanotubes [7,9-15]. These approaches have shown improved performance for Si materials in lithium-ion batteries. The nanostructured Si can provide sufficient intermediate space to withstand the large volume expansion involved with Li insertion and thus allow for expeditious elastic strain associated with the degradation upon cycling. Nevertheless, these Si nanostructures cannot be used commercially by now due to a difficult and expensive preparation process being mostly not compatible with standard silicon CMOS technology. Moreover, the formation of a solid/electrolyte interphase (SEI) at the interface layer between Si and the electrolyte is an obstacle for applications. Recently, it has been reported that metal silicide alloys composed of an active or inactive metal material can supply a capacity enhancement and better cycle life of Si-based electrodes [16-19]. It has been suggested that this is probably due to the provision of a better contact and a better mechanical stability for nanostructured Si electrodes.

In contrast to [16-19], we examine in this paper the potential of a fully CMOS-compatible technology for metal-coated 1-D nanowires on black silicon (nb-Si) using inductively coupled plasma (ICP) etching and successive coating by metal magnetron sputtering. As we have shown recently, ICP-etched black silicon has the potential to be a cost-competitive candidate for large-area nanostructured silicon anodes [20]. Metallic additive materials for Si-based electrodes are classified as active materials and inactive materials. The active materials can be influenced by Li ions, whereas the inactive materials are impervious to Li ions. For comparison and evaluation of both metallic additive materials, we used gold (Au) as a representative active metal material and copper (Cu) as an inactive metal material, respectively. We will show that also in the case of metal-coated nb-Si as anode, the electrochemical performance is better than in pristine nb-Si and discuss the influence of the metal thickness.

## Methods

### Materials and preparation

The nb-Si anode has been provided by direct ICP etching with a gaseous mixture comprised of SiF_6_ and C_4_F_8_ as discussed recently by us [20]. To achieve the deep nb-Si structure, the plasma etching process demands alternating plasma steps, including etching of Si and deposition of a passivation layer. This sequence etching process can generate the highly ordered nanostructured black Si [20,21]. ICP etching was carried out on polished 525 ± 25 μm-thick *n*-type Si (100)-oriented wafers with a resistivity of 1 to 5 Ω cm. To remove the fluorinated groups (-C_*x*_F_*y*_-) from the nb-Si surface induced by the ICP etching process, nb-Si was treated by heat. Afterwards, the wafer-scale nb-Si sample was broken into small pieces of suitable size of about 10 mm in diameter. Eventually, we prepared long nb-Si electrodes with a length of 28 μm and a diameter of approximately 730 nm. The average distance between the nanowires was about 1 μm. Au and Cu were deposited onto the surface of the nb-Si electrode in a sputter coater (108auto, Cressington Scientific Instruments Ltd., Watford, Hertfordshire, UK), equipped with a high-resolution thickness monitor system (MTM10, Cressington Scientific Instruments Ltd., Watford, Hertfordshire, UK). We prepared four metal (Au, Cu)-coated nb-Si electrodes with a thickness of 20 and 50 nm, respectively.

### Measurements

Electrochemical measurements of the nb-Si electrode were carried out by using Swagelok cells (Swagelok Company, Solon, OH, USA). Li metal foil was used as a counter/reference electrode. The backside of the nb-Si was contacted mechanically with Cu foil as a current collector without binder and electronic conductive material. Both cathode and anode electrodes were separated by a glass fiber filter paper (Whatman GF/B, Sigma-Aldrich, St. Louis, MO, USA), wetted with electrolyte. The electrolyte was 1 M LiPF_6_ in 1:1 (*w*/*w*) ethylene carbonate (EC) and diethyl carbonate (DEC) (Merck KGaA, Darmstadt, Germany). The cycling of the nb-Si electrode has been performed using the galvanostatic charge/discharge electrochemical technique with the Neware battery testing system (Neware Technology Limited., Shenzen, China). These cells were assembled and disassembled in an Ar-filled glove box (<0.1 ppm oxygen) at room temperature. The specific capacity of nb-Si was calculated based on the bare surface area. The discharge sequence represents lithiation due to the Si anode cell in this work. The surface morphology was characterized by field-emission scanning electron microscopy (FE-SEM, JSM-7401 F, JEOL Ltd., Tokyo, Japan) and energy-dispersive spectroscopy, and the X-ray diffraction (XRD) patterns were obtained by D/MAX-2200 V/PC (Rigaku, Tokyo, Japan) with Cu Kα radiation.

## Results and discussion

**Figure 1:**
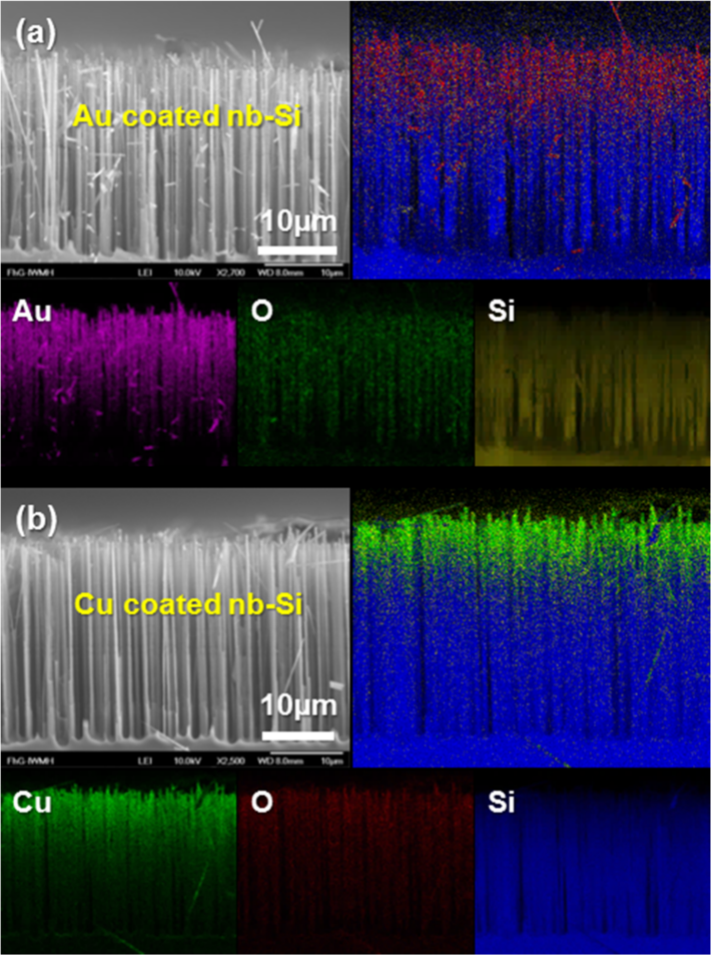
**Morphological characterization of the metal (Au, Cu)-coated nb-Si electrodes.** Cross-sectional SEM images of the **(a)** Au-coated and **(b)** Cu-coated nb-Si electrodes showing where the elemental maps were obtained and corresponding element mapping images of Au, Cu, O, and Si for metal (Au, Cu)-coated nb-Si electrodes. The upper right images of (a) and (b) show the summarized elemental signals.

Figure 1 shows SEM images of the as-prepared Au- and Cu-coated nb-Si electrodes, respectively. The vertically aligned Si structures were obtained by direct sequence ICP etching and magnetron sputtering technique on the Si substrate. As an original part of the Si substrate, the nb-Si ensures an effective charge transport through direct electric pathways. The element distribution of the metal coating was characterized by energy-dispersive X-ray spectroscopy (EDS) mapping. The Au and Cu images show the uniform lateral distribution of the metal along the nanowires with hardly any agglomeration. However, an intense signal of the metal was detected in the upper part, resulting from the fact that the metal elements can only penetrate into the top part of the nb-Si due to the physical nature of the sputtering process. The oxygen images are attributed to the surface oxidation covering nb-Si, and the Si images correspond to the morphology of nb-Si electrodes in the SEM images, respectively. These results confirm that the metal-coated nb-Si electrode is composed of silicon nanowires covered with a uniform Au and Cu coating with a small amount of native oxide on the nb-Si surface.

**Figure 2:**
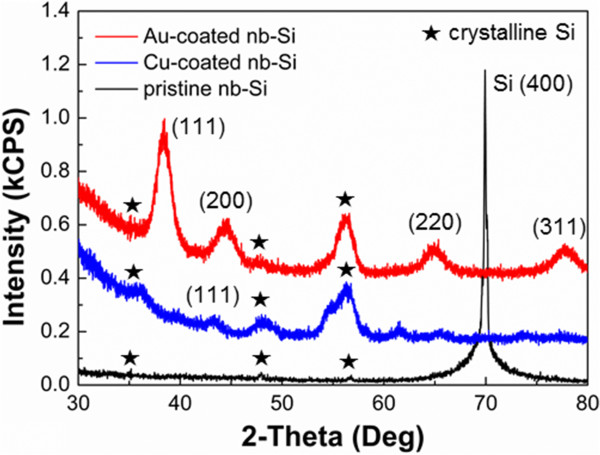
Comparison of X-ray diffraction (XRD) patterns of the metal (Au, Cu)-coated and pristine nb-Si electrode.

To determine the structural properties of metal-coated nb-Si, XRD patterns of the metal-coated nb-Si were compared with those of the pristine nb-Si. Figure 2 shows XRD patterns of Cu-coated, Au-coated, and pristine nb-Si. The XRD patterns of metal-coated nb-Si indicate that diffraction peaks are associated with crystalline structures of Au (111), (200), (220), and (311) regarding Au-coated nb-Si, and Cu (111) regarding Cu-coated nb-Si, respectively. In addition, peaks of crystalline silicon were detected, such as (111), (220), and (311). The intensity of the Si (400) peak at ca. 70 2*θ* is high compared to others induced by the large crystalline Si substrate.

**Figure 3:**
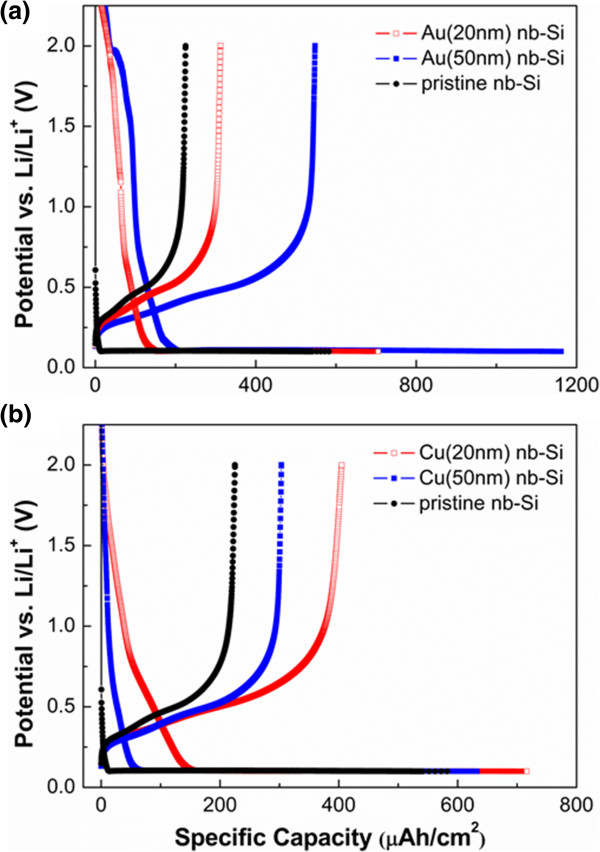
**Initial galvanostatic charge/discharge curves of the nb-Si electrode.** The voltage range is between 0.1 and 2.0 V (vs. Li/Li^+^) at a constant current density of 50 μA cm^-2^. The first cycle of the **(a)** Au-coated and **(b)** Cu-coated nb-Si electrode compared with that of the pristine nb-Si electrode.

**Figure 4:**
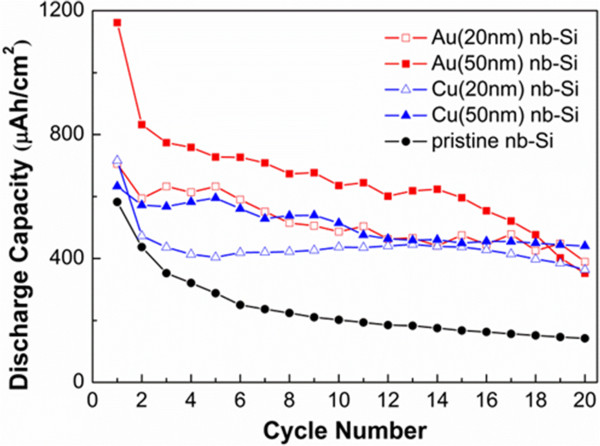
**Discharge capacities versus cycle number for the nb-Si electrodes.** Plot of the discharge capacities for the Au-coated (red squares) and Cu-coated (blue triangles) nb-Si electrodes compared with the pristine (black circles) nb-Si electrode. The performance cycle condition is the same as for Figure 3.

To investigate the cycling performance of metal (Au, Cu)-coated nb-Si electrodes, we used the galvanostatic electrochemical technique at a constant current density of 50 μA cm^-2^. The voltage range was from 0.1 to 2.0 V (vs. Li/Li^+^). Figure 3a shows the first charge/discharge curves of 20- and 50-nm Au-coated nb-Si. The first discharge capacity reached 705.5 and 1,161.5 μAh cm^-2^ with a coulombic efficiency of 44.3% and 47.2%, respectively. This is significantly higher than that of pristine nb-Si. Moreover, the discharge capacity of Au(50)-coated nb-Si is higher than that of Au(20)-coated nb-Si. This is because Au could contribute to form Au-Li-Si alloys as an active material for lithium-ion battery systems. In comparison, the first discharge capacity of Cu(20)- and Cu(50)-coated nb-Si was 716.1 and 632.7 μAh cm^-2^, giving a coulombic efficiency of 56.6% and 47.9%, respectively, in Figure 3b. This is still slightly higher compared to pristine nb-Si as well. However, the increase is not as strong as in the case of the Au coating since Cu is an inactive material. In addition, the discharge capacity of the thin Cu(20) coating is slightly higher than that of the thick Cu(50) coating in contrast to the Au coatings. Regarding the influence of the different thicknesses of Au and Cu, the Au coating shows a significant enhancement in capacity with increasing thickness of Au. In contrast, the thickness of the Cu coating has hardly any influence on the specific capacity at the first cycle. Note that all prepared metal-coated nb-Si electrodes show a relative low coulombic efficiency of around 50%, mainly resulting from the slow reaction paths along the huge specific surface area. Figure 4 shows the discharge capacity of the metal-coated nb-Si electrode versus cycle number at a constant current density of 50 μA cm^-2^. Whereas pristine nb-Si has a remaining capacity of only 24% after 20 cycles, the 50-nm Cu-coated samples still have about 70% (Table 1).

**Table 1:** Discharge capacity and capacity retention of pristine nb-Si and metal (Au, Cu)-coated nb-Si electrodes

**Sample**	**1st (μAh cm**^**-2**^**)**	**20th (μAh cm**^**-2**^**)**	**Capacity retention (%)**
Pristine nb-Si	582.3	142.3	24.4
Au(20)-coated nb-Si	705.3	389.0	55.2
Au(50)-coated nb-Si	1,161.5	352.1	30.3
Cu(20)-coated nb-Si	716.1	363.3	50.7
Cu(50)-coated nb-Si	632.7	440.4	69.6

Constant current density of 50 μA cm^-2^, potential range from 0.1 to 2.5 V (vs. Li/Li^+^).

**Figure 5:**
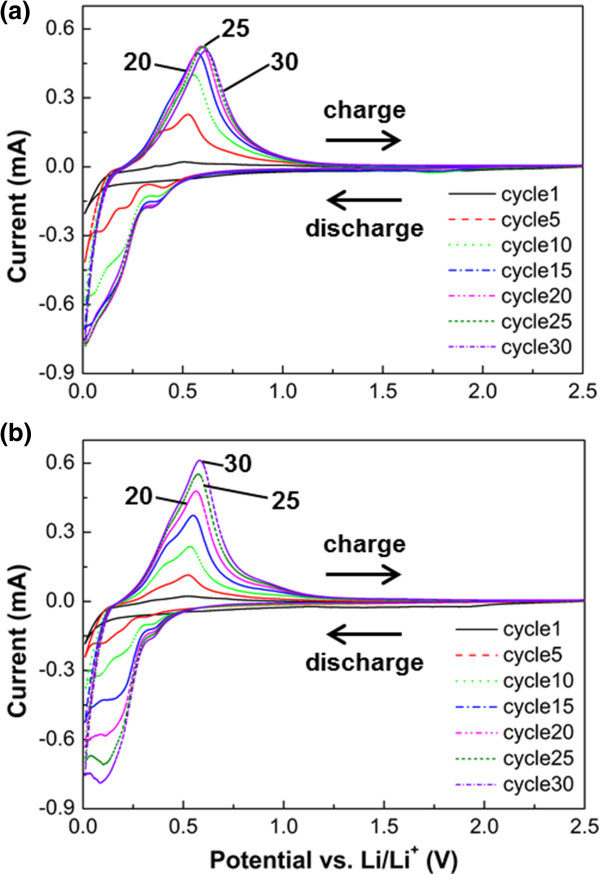
**Cyclic voltammograms for the metal (Au, Cu)-coated nb-Si electrodes.** The cycle voltage range is between 0.01 and 2.5 V (vs. Li/Li^+^) at scan rate of 0.5 mV s^-1^. Plot of cyclic voltammograms for the **(a)** Au-coated and **(b)** Cu-coated nb-Si electrodes.

**Figure 6:**
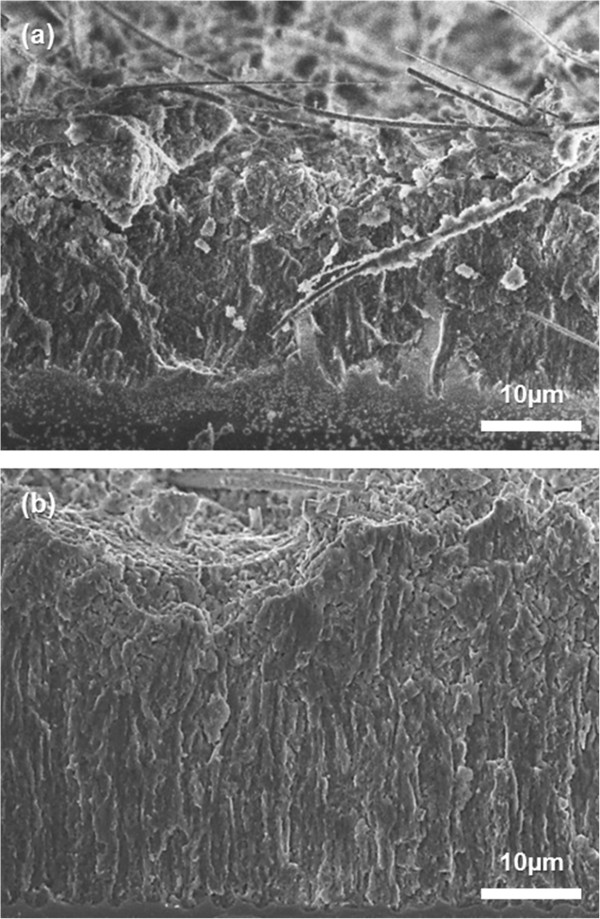
**Cross-sectional SEM images of the nb-Si electrode after 50 cycles.** The cells were charged to 2.0 V (vs. Li/Li^+^). **(a)** Pristine nb-Si and **(b)** Cu(50)-coated nb-Si electrodes.

Our electrochemical cycling results show that the metal coating can improve not only the discharge capacity but also the capacity retention compared to pristine nb-Si, confirming previous results on other silicon nanostructures [9,16-18] and extending it to CMOS-compatible processes. Therefore, we further investigated with cyclic voltammetry (CV) the Au-coated and Cu-coated nb-Si electrodes in the potential range between 0.01 and 2.5 V (vs. Li/Li^+^) at a scan rate of 0.5 mV s^-1^ (as shown in Figure 5). In Figure 5a for the Au-coated nb-Si, a characteristic peak at about 0.15 V indicates the onset of formation of the Li-Si alloy during discharging, and a peak at about 0.5 V implies the Li-Si de-alloying during charging. These peaks have shifted to a slightly higher potential of approximately 0.25 V and approximately 0.6 V, respectively, during cycling. In the same manner, the Cu-coated nb-Si electrode shows a similar phenomenon at the first cycle as shown in Figure 5b. However, the following cycles show a different behavior. In the case of the Au-coated nb-Si electrode, the current peak increased rapidly within about 15 cycles since Au as an active material leads to an increasing specific capacity by forming Au-Li-Si alloys, as mentioned before, and then stagnated or decreased a little after subsequent cycles. In contrast, the current peak of the Cu-coated nb-Si electrode increased gradually up to the 30th cycle. Note that the Au metal is more active with Li-Si alloying/de-alloying than the Cu metal at the beginning of cycles, resulting in the initial high specific capacity. To further verify the improved behavior of the morphology and volume changes of metal-coated nb-Si, particularly the Cu-coated samples, we performed SEM analysis of the metal-coated nb-Si at a full delithiation after 50 cycles compared to a pristine one (as shown in Figure 6). Most of the metal-coated nb-Si retained their original shape without cracking or fracturing. The metal-coated nb-Si show only slight deformations and volume changes, resulting from the amorphization during continuous cycling.

## Conclusions

We developed a fully CMOS-compatible technology for the fabrication of Au- and Cu-coated nb-Si anodes for microbatteries with a similar capacity and stability compared to conventional carbon-based technologies. The silicon nanowire anodes are prepared by ICP etching method of silicon and successive magnetron sputtering of metals. In particular, Cu-coated (50 nm) nb-Si has the lowest capacity fading and a stable capacity retention during cycling. The difference of the electrochemical properties of Cu and Au can be explained by the better adhesion of copper on silicon in line with recent *in situ* experiments [22]. We believe further improvements of the capacity can be made by continuing to optimize the electrolyte/anode interface.

## Abbreviations

CMOS: complementary metal-oxide semiconductor; ICP: inductively coupled plasma; SEI: solid/electrolyte interphase.

## Competing interests

The authors declare that they have no competing interests.

## Authors’ contributions

GL designed the experiments, performed the fabrication and the measurements, and managed the interpretation of data. GL and SLS drafted the manuscript. RBW supervised the project. All authors discussed the results and commented on the manuscript. All authors read and approved the final manuscript.

## References

[B1] Roberts M Johns P Owen J Brandell D Edstrom K El Enany G Guery C Golodnitsky D Lacey M Lecoeur C Mazor H Peled E Perre E Shaijumon MM Simon P Taberna PL 3D lithium ion batteries—from fundamentals to fabrication J Mater Chem 2011 21 9876 9890 10.1039/c0jm04396f

[B2] Scrosati B Garche J Lithium batteries: status, prospects and future J Power Sources 2010 195 2419 2430 10.1016/j.jpowsour.2009.11.048

[B3] Ellis BL Knauth P Djenizian T Three-dimensional self-supported metal oxides for advanced energy storage Adv Mater 2014 26 3368 3397 10.1002/adma.201306126 24700719

[B4] Huggins RA Lithium alloy negative electrodes J Power Sources 1999 81 13 19

[B5] Szczech JR Jin S Nanostructured silicon for high capacity lithium battery anodes Energy Environ Sci 2011 4 56 72 10.1039/c0ee00281j

[B6] Beaulieu L Hatchard T Bonakdarpour A Fleischauer M Dahn J Reaction of Li with alloy thin films studied by in situ AFM J Electrochem Soc 2003 150 A1457 A1464 10.1149/1.1613668

[B7] Chan CK Peng H Liu G McIlwrath K Zhang XF Huggins RA Cui Y High-performance lithium battery anodes using silicon nanowires Nat Nanotechnol 2007 3 31 35 18654447 10.1038/nnano.2007.411

[B8] Li H Huang X Chen L Wu Z Liang Y A high capacity nano Si composite anode material for lithium rechargeable batteries Electrochem Solid State Lett 1999 2 547 549 10.1149/1.1390899

[B9] Shin H Corno JA Gole JL Liu M Porous silicon negative electrodes for rechargeable lithium batteries J Power Sources 2005 139 314 320 10.1016/j.jpowsour.2004.06.073

[B10] Kang D Corno JA Gole JL Shin H Microstructured nanopore-walled porous silicon as an anode material for rechargeable lithium batteries J Electrochem Soc 2008 155 A276 A281 10.1149/1.2836570

[B11] Cui L Ruffo R Chan CK Peng H Cui Y Crystalline-amorphous core-shell silicon nanowires for high capacity and high current battery electrodes Nano Lett 2008 9 491 495 19105648 10.1021/nl8036323

[B12] Kim H Han B Choo J Cho J Three‒dimensional porous silicon particles for use in high‒performance lithium secondary batteries Angew Chem Int Edit 2008 120 10305 10308 10.1002/ange.200804355 19016293

[B13] Song T Xia J Lee J Lee DH Kwon M Choi J Wu J Doo SK Chang H Park WI Zang DS Kim H Huang Y Hwang K Rogers A Paik U Arrays of sealed silicon nanotubes as anodes for lithium ion batteries Nano Lett 2010 10 1710 1716 10.1021/nl100086e 20369889

[B14] Park M Kim MG Joo J Kim K Kim J Ahn S Cui Y Cho J Silicon nanotube battery anodes Nano Lett 2009 9 3844 3847 10.1021/nl902058c 19746961

[B15] Astrova E Fedulova G Smirnova I Remenyuk A Kulova T Skundin A Porous silicon based negative electrodes for lithium ion batteries Tech Phys Lett 2011 37 731 734 10.1134/S1063785011080037

[B16] Sethuraman VA Kowolik K Srinivasan V Increased cycling efficiency and rate capability of copper-coated silicon anodes in lithium-ion batteries J Power Sources 2011 196 393 398 10.1016/j.jpowsour.2010.06.043

[B17] Ossei‒Wusu E Cojocaru A Hartz H Carstensen J Föll H Silicon nanowires made via macropore etching for superior Li ion batteries Phys Status Solidi A 2011 208 1417 1421 10.1002/pssa.201000031

[B18] Vlad A Reddy AL Ajayan A Singh N Gohy JF Melinte S Ajayan PM Roll up nanowire battery from silicon chips Proc Natl Acad Sci U S A 2012 109 15168 15173 10.1073/pnas.1208638109 22949696 PMC3458382

[B19] Thakur M Isaacson M Sinsabaugh SL Wong MS Biswal SL Gold-coated porous silicon films as anodes for lithium ion batteries J Power Sources 2012 205 426 432

[B20] Lee G Schweizer SL Wehrspohn RB Electrochemical characteristics of plasma-etched black silicon as anode for Li-ion batteries J Vac Sci Technol A 2014 32 061202

[B21] Volland B Shi F Hudek P Heerlein H Rangelow IW Dry etching with gas chopping without rippled sidewalls J Vac Sci Technol B 1999 17 2768 2771 10.1116/1.591061

[B22] McDowell MT Woo Lee S Wang C Cui Y The effect of metallic coatings and crystallinity on the volume expansion of silicon during electrochemical lithiation/delithiation Nano Energy 2012 1 401 410 10.1016/j.nanoen.2012.03.004

